# Expression Analysis of let-7a-5p and miR-21-3p in Extracellular Vesicles Derived from Serum of NSCLC Patients

**DOI:** 10.3390/biomedicines13092060

**Published:** 2025-08-24

**Authors:** Dian Jamel Salih, Katrin S. Reiners, Domenico Loizzi, Nicoletta Pia Ardò, Teresa Antonia Santantonio, Francesco Sollitto, Gunther Hartmann

**Affiliations:** 1 Department of Medical and Surgical Sciences, University of Foggia, Via Napoli, 121, 71122 Foggia, Italy; 2 Department of Anatomy, Biology and Histology, College of Medicine, University of Duhok, Zakho Way, Duhok 42001, Iraq; 3 Institute of Clinical Chemistry and Clinical Pharmacology, University Hospital Bonn, 53127 Bonn, Germany; kreiners@uni-bonn.de (K.S.R.); gunther.hartmann@uni-bonn.de (G.H.); 4 Institute of Thoracic Surgery, Department of Medical and Surgical Sciences, University of Foggia, 71122 Foggia, Italy; domenico.loizzi@unifg.it (D.L.); nicoletta.ardo@gmail.com (N.P.A.); francesco.sollitto@unifg.it (F.S.); 5 Infectious Diseases Unit, Department of Clinical and Surgical Sciences, University of Foggia, 71122 Foggia, Italy; teresa.santantonio@unifg.it

**Keywords:** lung cancer, extracellular vesicles, miRNA, let-7a, miR-21

## Abstract

**Background/Objectives**: Despite the significant advancements made in the diagnosis of lung cancer, the traditional diagnostic methods remain limited because they are often invasive, expensive, and not suitable for regular screening, creating a need for more accessible and non-invasive alternatives. In this context, the analysis of miRNAs in EVs and free circulating microRNA may be used as liquid biopsies in lung cancer to identify individuals at risk. This study aimed to compare miRNA profiles in the serum and EVs derived from lung cancer patients by focusing on Let-7a-5p and miR-21-3p. **Materials and Methods:** Serum and EVs were isolated from lung cancer patients and healthy controls. EVs were characterized using nanoparticle tracking analysis, electron microscopy, and Western blotting for surface markers (CD63, CD81, TSG101). Total miRNA levels were quantified in the serum and EVs, and specific miRNAs (hsa-let-7a-5p and hsa-miR-21-3p) were analyzed using RT-qPCR. Statistical analysis evaluated miRNA expression across clinicopathological features, including age, gender, smoking status, tumor stage, cancer type, and EGFR mutation status. **Results:** Total miRNA levels were significantly enriched in EVs compared to the serum. Let-7a-5p was downregulated in EVs from patients with advanced-stage lung cancer (Stage III–IV) compared to those with early-stage cancer and controls (*p* < 0.05), while no differences were observed in the serum. Conversely, miR-21-3p was significantly upregulated in EVs and serum from advanced-stage patients (*p* < 0.01) and in adenocarcinoma compared to squamous cell carcinoma (*p* < 0.05). No significant differences were observed for age, gender, or smoking status. **Conclusions:** Our findings highlight the differential expression of miRNAs in EVs and the serum, emphasizing the diagnostic potential of EV-associated Let-7a-5p and miR-21-3p in lung cancer. These results suggest that EVs are a more robust source for miRNA biomarkers compared to the serum.

## 1. Introduction

Lung cancer is still the leading cause of cancer deaths worldwide due to its late diagnosis. Early detection significantly improves prognosis by reducing mortality and enhancing survival rate, adding to successful treatment by focusing on detecting asymptomatic patients. However, the diagnostic techniques currently available have major limitations, especially in the early detection of cancer [1].

CT has a high false-positive rate, indicating the presence of cancer when the reality is otherwise, and sputum cytology is only of limited sensitivity in locating minute lung tumors that are in the peripheral regions of the lungs. Therefore, these methods are not particularly effective in the early detection of lung cancer [2]. Considering these limitations, there is an urgent need for non-invasive and reliable biomarkers for the early-stage detection of lung cancer.

Recent extensive studies have identified microRNAs (miRNAs) as potential biomarkers for the diagnosis, prognosis, and targeted therapy of lung cancer. miRNAs are a class of highly conserved small non-coding RNAs, varying between 18 and 25 nucleotides in length [3], that modulate gene expression at the post-transcriptional level by binding to the complementary 3′ untranslated region (3′UTR) of target mRNAs, thereby facilitating mRNA degradation or inhibiting protein synthesis [4].

Bioinformatics algorithms predict that miRNAs regulate more than 60% of the protein-coding genes in the human genome. One miRNA can regulate numerous target mRNAs, just as one mRNA is regulated by several miRNAs. Thus, miRNAs are important in many biological processes, including gene regulation, apoptosis, hematopoietic development, and the maintenance of cell differentiation [5,6].

Multiple studies indicate the dysregulation of miRNAs involved in the pathogenesis of different cancers, including lung cancer, through the regulation of various biological processes such as cell proliferation [7], differentiation [8], angiogenesis [9], and apoptosis [10]. Specific miRNAs have oncogenic or tumor-suppressing potential, and their altered expression profiles in cancer patients serve as diagnostic biomarkers [11].

Previous investigations have reported that a variety of differentially expressed miRNAs in lung cancer may have potential as diagnostic biomarkers. For example, studies have pointed out that several miRNAs such as miR-21, miR-106a, miR-10b, miR-328, miR-155, let-7, and miR-103 are usually dysregulated in lung cancer patients [12,13,14].

These miRNAs have been found in several body fluids, such as the plasma and serum, and thus are accessible for non-invasive diagnostic tests. Bai et al. [13] found that miR-21 was significantly overexpressed in the serum of NSCLC patients compared with healthy controls, indicating a possible role as a diagnostic biomarker. Similarly, Xue et al. [15] reported elevated levels of miR-21 and miR-155 in the plasma of lung cancer patients, correlating with a poor prognosis.

MicroRNAs are unstable in body fluid and susceptible to degradation by ribonucleases (RNases) present in the bloodstream, which can affect their reliability as diagnostic biomarkers. Due to their instability, researchers are investigating alternative sources of miRNA that are more stable and less susceptible to degradation [16]. Recently, extracellular vesicles (EVs) have gained increasing attention in biomedical research as a promising solution to this problem due to the cargo composition of miRNAs in EVs and their stability in body fluids [6].

EVs are small, heterogeneous, membrane-bounded vesicles released by different cell types into the extracellular environment, contributing to intercellular communication. EVs are enriched with proteins, lipids, and nucleic acids, including DNA, mRNA, and miRNA. Based on their size and biogenesis, they have been categorized into exosomes, microvesicles, and apoptotic bodies [17]. The miRNA cargo of EVs reflects the molecular signature of their parental cell, hence becoming attractive candidates for the non-invasive diagnosis of cancer. Also, the miRNA packaged within EVs is more stable and reliable than free miRNA in plasma or serum because the phospholipid bilayer encapsulated in EVs protects them from the RNases degradation in body fluids [18].

Several studies have investigated the miRNA profiles of EVs derived from lung cancer patients and proved their potential in diagnostics. Rabinowits et al. [19] found that the miRNA profiles of EVs derived from plasma and tumor tissues of NSCLC were distinct from those of healthy individuals, with upregulated levels of 12 specific miRNAs, including miR-21, miR-210, and miR-223, meaning that the profile of plasma exosomal miRNAs parallels that of tumor-derived miRNAs. Similarly, Wu et al. demonstrated that serum EV miRNA-96 expression was higher in lung cancer patients than in controls and displayed a correlation with the stage of the cancer [20]. Although EV miRNA has tremendous potential, further studies are required to confirm the above findings and to develop reliable diagnostic assays.

However, there are still technical challenges in the isolation and characterization of EVs, necessitating the need for standardized protocols to ensure reproducibility and accuracy in miRNA detection. The aim of this study was to investigate the microRNA profile in EVs isolated from lung cancer by quantifying and comparing the total miRNA levels in the serum and EVs to assess their diagnostic potential. Subsequently, we focused on two specific miRNAs previously reported to be dysregulated in serum and lung cancer tissues, namely miR-let-7a, functioning as a tumor suppressor, and miR-21, identified as oncogenic, which were isolated and analyzed separately in both serum and EVs.

## 2. Materials and Methods

### 2.1. Patients’ Samples

Whole blood samples from 37 patients with lung cancer and 37 healthy controls who showed no evidence of cancer or other diseases were collected before treatment at Foggia Hospital in the south of Italy. Venous blood samples (3 mL) were collected in coagulation-promoting tubes. The samples were allowed to coagulate in an upright position for 30 min without being disturbed and then centrifuged at 2500× *g* for 10 min at 20 °C. The top layer containing serum was carefully transferred to a 15 mL reaction tube and centrifuged at 3200× *g* for 20 min at 20 °C. Finally, the serum was transferred into a fresh 1.5 mL reaction tube or cryotube without disturbing the pellet and filtered with a 0.45 μM filter, and the serum samples were stored at −80 °C until needed.

### 2.2. Isolation of EVs by Size Exclusion Chromatography

EVs were isolated from serum samples by means of size exclusion chromatography (SEC) using sepharose-based qEV columns (iZON Science, Christchurch, New Zealand) according to the manufacturer’s recommendations and previously published methods [18].

Briefly, the columns were removed from a 4 °C environment and slowly adapted to room temperature, the 20% ethanol storage solution was removed, and the column was flushed one to two times with 10 mL of PBS. The serum samples were thawed on ice and centrifuged for 10 min at 1500× *g* at 10 °C, and the supernatant was transferred to new Eppendorf tubes and subjected to another centrifugation for 10 min at 10,000× *g* at 10 °C. Each serum sample was transferred to a new Eppendorf tube and stored at −80 °C or processed directly. For frozen samples, an additional centrifugation at 10,000× *g* for 10 min at 10 °C was performed after thawing and immediately prior to SEC to remove aggregates potentially formed during storage. A 0.5 mL aliquot of serum was applied to the qEV column, and the flow-through was discarded. Then, 3900 µL of Hank’s balanced salt solution (HBSS) was applied, and the flow-through was discarded. Another 3 × 500 μL of HBSS was applied and the flow-through was collected and protease inhibitor was added. Finally, the 1.5 mL samples of the collected EVs were subjected to ultracentrifugation at 130,000× *g* for 2.5 h at 4 °C. The supernatant was carefully removed, leaving 115 μL. This was used to carefully resuspend the pellet by pipetting for 5 min. EV suspensions were kept at −80 °C until further application.

### 2.3. Nanoparticle Tracking Analysis

The particle size distribution and concentration of EVs were assessed using nanoparticle tracking analysis (NTA), following established protocols [3]. Briefly, EV suspensions were diluted in HBSS buffer to obtain a final measurement volume of 1 mL, with dilution ratios ranging between 1:50 and 1:2000. The working concentration was optimized by ensuring a particle count of approximately 140–200 per frame. Each analysis consisted of two measurement runs, scanning 11 distinct positions, and capturing 30 frames at each position. Data acquisition was carried out under standardized conditions, including automatic focus adjustment, a fixed camera sensitivity of 79, a shutter speed of 70, auto-detected scattering intensity, and a chamber temperature of 24 °C. Recorded videos were processed using ZetaView Software (version 8.05.11 SP1, Particle Metrix, Meerbusch, Germany) on the ZetaView PMX-120 instrument, applying the following analytical thresholds: a minimum area of 5, a minimum brightness of 30, and a maximum area of 1000. The system hardware consisted of a CMOS camera and an integrated 40 mW, 488 nm laser source.

### 2.4. Transmission Electron Microscopy (TEM)

Transmission electron microscopy (TEM) was employed to confirm and analyze the morphology and size of EVs. For sample preparation, 3 μL of the EV suspension was placed onto glow-discharged copper grids coated with pioloform carbon and left to adsorb for 5 min. The grids were subsequently rinsed twice with drops of deionized distilled water. Negative staining was carried out by applying two successive 4 μL drops of 0.5% aqueous uranyl acetate. After excess stain was carefully removed with filter paper, the grids were air-dried prior to imaging. The vesicles were visualized using a JEOL JEM-1400 transmission electron microscope operated at 80 kV. Digital images were acquired with a Megaview III camera system and processed using iTEM software 5.2.

### 2.5. Western Blotting

Proteins from the extracellular vesicles were extracted using 1 × RIPA lysis buffer composed of 50 mM Tris-HCl (pH 7.6), 150 mM NaCl, 1% Triton X-100, 0.5% sodium deoxycholate, 0.1% SDS, and supplemented with a protease and phosphatase inhibitor cocktail. The protein was estimated using the BCA protein assay kit (ThermoFisher, Waltham, MA, USA, Cat#: 23227), and 5X Laemmli buffer was added to the lysate solution. Then, equal volumes of protein (50 µg) for each sample were prepared by separating them via 10% SDS-PAGE and then transferring them onto 0.45 µM nitrocellulose membranes (Sigma-Aldrich, ST. Louis, MO, USA, GE10600003). The membranes were blocked in 5% BSA in TBS-T for an hour at room temperature after being rinsed four times for five minutes each time with TBS buffer, followed by immunoblotting with primary antibodies of interest. The following antibodies were used: CD9 (1:1000. rabbit monoclonal, Abcam, Cambridge, UK, Cat#: ab92726), CD81 (1:1000. mouse monoclonal, Cat#: sc-166029), flotillin-1 (1:5000. mouse monoclonal, BD Biosciences, San Jose, CA, USA, Cat#: 610821), and TSG101 (1:5000. mouse monoclonal, Santa Cruz, CA, USA, Cat#: sc-7964). Membranes were again washed another four times for five minutes with TBS-T, and HRP-conjugated secondary anti-mouse antibody (1:10,000. mouse monoclonal, Cell signaling, Danvers, MA, USA, Cat#: 7076) was added for 1 h at room temperature. The detection of protein bands was carried out with an enhanced chemiluminescence (ECL) reagent (GE Healthcare Life Sciences, Chicago, IL, USA), and the resulting signals were captured and analyzed using the Odyssey Fc imaging system (LI-COR Biosciences, Lincoln, NE, USA).

### 2.6. Isolation of miRNAs

The EVs, after purification, were transferred to RNase-free tubes. Total RNA, including miRNA, from 100 µL of EVs and 200 µL of serum was extracted utilizing the QIAGEN miRNeasy Micro Kit (Qiagen, Hilden, Germany) in accordance with the manufacturer’s instructions. RNA was eluted in 30 µL of RNase-free water provided with the kit. The NanoDrop One spectrophotometer (Thermo Fisher Scientific, Waltham, MA, USA) was employed to assess the concentration and purity of the eluted RNA. RNA purity was evaluated with the absorbance ratio OD260/OD280.

### 2.7. miRNAs Reverse Transcription and Expression by qRT-PCR

Following miRNA extraction, miRNA quantification was performed by using RNA samples with equal volumes for cDNA synthesis using miRCURY LNA RT kit according to the manufacturer’s protocol (Qiagen, Hilden, Germany). Quantitative RT-PCR (qRT-PCR) was performed by using a QuantStudio 5 (ThermoFisher Scientific, Waltham, MA, USA) with miRCURY LNA SYBR Green PCR Kit (Qiagen, Hilden, Germany). Among the various target miRNAs, we focused on two microRNAs that have previously been reported as being altered in serum or lung cancer tissue samples: miR-let-7a-5p, identified as a tumor suppressor miRNA, and miR-21-3p, classified as an oncogenic miRNA. miR-103a-3p was selected as the internal control because it is consistently expressed in healthy populations and has not been reported to show alterations in lung cancer (Table 1). The PCR reactions were carried out in duplicate with 60 cycles of denaturation (10 s at 95 °C), annealing (60 s at 56 °C), and elongation (30 s at 54 °C) after an initial enzyme activation (2 min at 95 °C). The expression levels of EV miRNA in the serum and free circulating miRNA of patients with lung cancer were compared with those of healthy controls. The expression levels of the target miRNAs were normalized to the miR-103a, and the calculation was performed using the comparative 2−ΔΔCt method.

Normalization was performed using miR-103a, which has been reported to show stable expression across diverse tissue types and pathological conditions, including cancer. In a previous work from our group, systematic screening identified miR-103a and miR-484 as reliable endogenous reference miRNAs in serum and extracellular vesicle studies [3,17]. In our present dataset, miR-103a expression did not differ significantly between NSCLC patients and controls, supporting its use as an internal normalizer.

### 2.8. Statistical Analysis

All statistical analyses were performed using SPSS version 22.0 (IBM, Armonk, NY, USA), while figures and graphs were generated with GraphPad Prism version 10.1.2 (GraphPad Software, San Diego, CA, USA). To assess the adequacy of this sample size, we conducted a post hoc power analysis using the two-sample t-test model. Assuming a medium-to-large effect size (Cohen’s d = 0.7) and a significance level of 0.05, the analysis indicated a statistical power of approximately 0.84, which exceeds the commonly accepted threshold of 0.80. The results are expressed as average ± SD. One-way ANOVA and Student’s t-test were used to analyze the statistical significance of the differences between the groups. A threshold of *p* < 0.05 was considered statistically significant.

## 3. Results

### 3.1. Participant Characteristics

In the present study, from January 2023 to December 2023, serum samples from a total of 37 lung cancer patients who visited the Thoracic Surgery Department at the A.O.U. Polyclinic in Foggia, Italy, and 37 healthy controls (HC) were investigated. Among them were 31 patients with adenocarcinoma and 6 with squamous cell carcinoma. In lung cancer patients, samples were prospectively collected at a time prior to any treatment. The clinicopathological characteristics of the participants are shown in Table 2.

### 3.2. EVs Are Significantly Enriched in NSCLC Patient Serum

Circulating EVs were isolated from the serum of patients with lung cancer and healthy controls by means of size exclusion chromatography (SEC). The EV-enriched fractions were characterized by their size, morphology, concentration, and protein markers.

**Figure 1:**
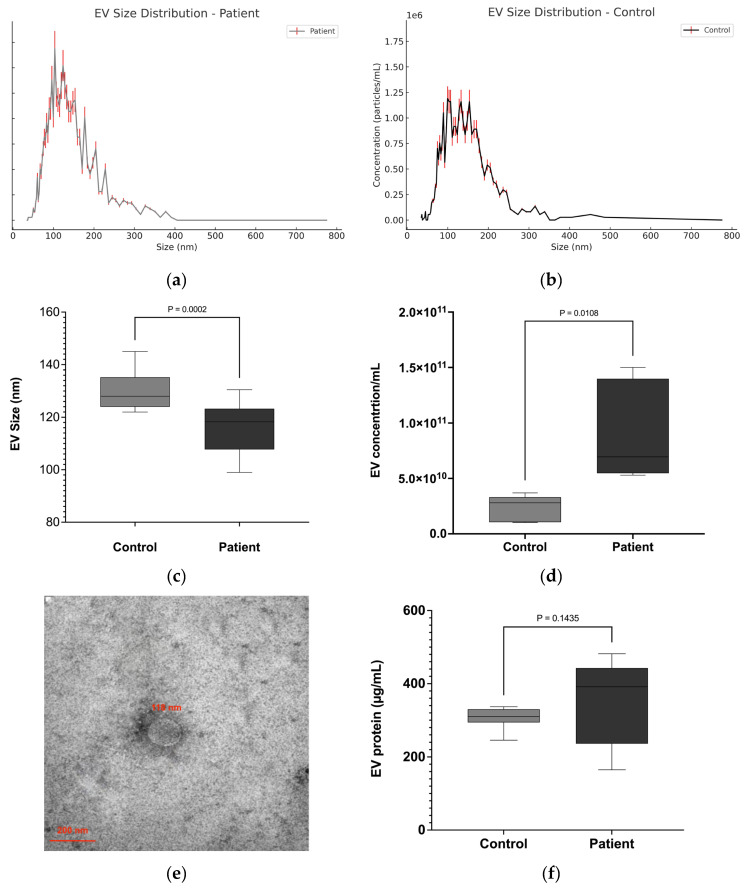
Characterization of EVs derived from lung cancer patients. NTA was used to analyze the size and concentration of EVs. (**a**–**c**) EV size: Representative measurements of lung cancer patient (**a**) and healthy control (**b**) EVs showing the size range of the isolated EVs. (**c**) Median sizes of EVs from lung cancer patients (116 ± 9.91 nm) and healthy individuals (128 ± 7.63 nm). (**d**) EV concentrations in lung cancer patients (7.99 ± 0.85 × 10^10^ particles/mL) and in healthy controls (3.58 ± 0.42 × 10^10^ particles/mL) particles/mL. (**e**) TEM image of EVs derived from patient serum. EVs, negatively stained with 2% uracyl acetate after removal of moisture, showed the expected size and shape of small EVs (30–150 nm in diameter). (**f**) EV protein quantification. *n* = 37.

The NTA results showed that the diameter of EVs isolated from the serum of patients and healthy individuals primarily ranged between 50 and 200 nm in diameter (Figure 1a–c). The median size of EVs in lung cancer patients was significantly smaller (116 ± 9.91 nm) compared to healthy controls (128 ± 7.63 nm) (Figure 1c). Quantification of EVs by NTA revealed that the concentration of EVs in lung cancer patients (7.99 ± 0.85 × 10^10^ particles/mL) was significantly higher compared to healthy controls (3.58 ± 0.42 × 10^10^ particles/mL) (*p* ≤ 0.01), representing an approximately 2.2-fold increase (Figure 1d). TEM analyses revealed a majority of vesicles in the expected size range of 70–150 nm, which is characteristic of small EVs, from both NSCLC patient serum and that of healthy controls (Figure 1e). In addition, the protein concentration of the EV samples was evaluated. No significant differences were observed regarding the protein concentration of EVs from patient (349 ± 25.7 µg/mL) or HC serum (307 ± 28.43 µg/mL) (Figure 1f). However, the higher protein concentrations of the patient EV samples reflect the higher number of EVs isolated from 0.5 mL of patient serum.

### 3.3. Western Blot Analysis Confirms Purity of EVs Isolates

**Figure 2:**
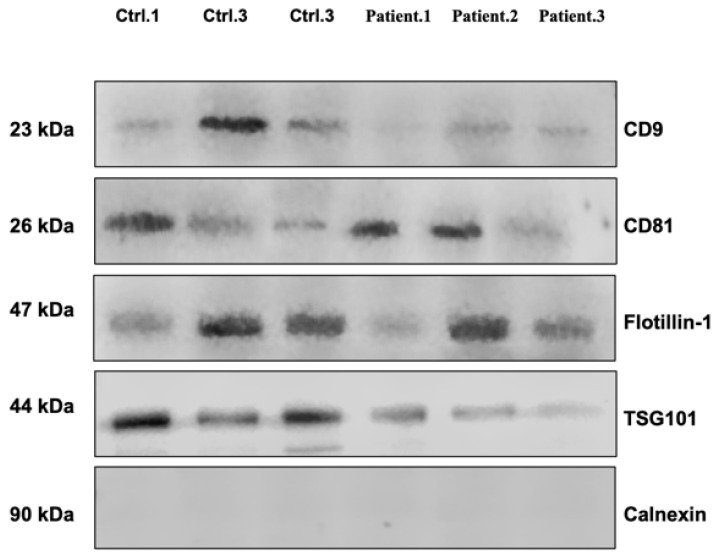
Immunoblotting assay for EV markers detected CD9, CD81, Flotillin-1 and TSG101 in EVs derived from lung cancer patients and healthy controls. Calnexin was used as a negative control.

To ensure the successful enrichment and purity of EVs after SEC isolation, EVs were analyzed by means of Western blot to confirm the presence of EV markers and absence of proteins indicating contamination. In accordance with the Minimal Information for Studies of EVs 2024 (MISEV) [21], several known EV markers, including CD9, CD81, Flotillin-1, and TSG101, were observed in the purified serum EVs of patients with lung cancer and healthy individuals. Meanwhile, the endoplasmic reticulum marker calnexin was absent in our isolated EV samples, indicating their purity (Figure 2).

### 3.4. miRNAs Are Targeted into EVs

**Figure 3:**
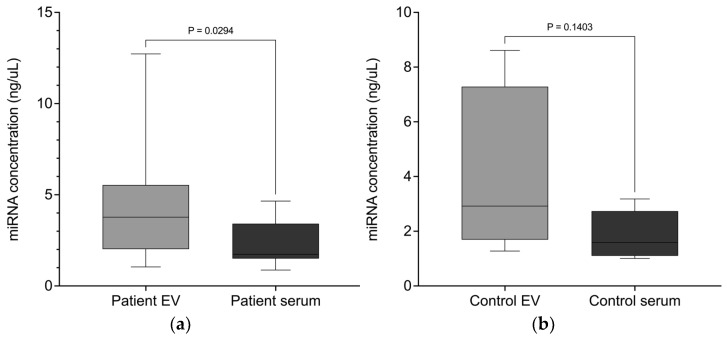
miRNAs are enriched in EVs after normalization to serum input volume. Box plots illustrate the distribution of normalized total miRNA concentrations in extracellular vesicles (EVs) and matched free-circulating serum fractions from lung cancer patients and healthy controls. miRNA concentrations were calculated based on original serum input volumes. Each plot shows the median with the interquartile range. (**a**) In lung cancer patients, EVs yielded significantly higher miRNA concentrations normalized to serum (mean: 4.71 ng/µL) than serum-derived free-circulating miRNA (2.39 ng/µL, *p* = 0.0294). (**b**) Among healthy controls, EV miRNA concentration (mean: 4.18 ng/µL) was also higher than in the serum (1.85 ng/µL), but the difference was not statistically significant (*p* = 0.1403).

In the present study, we isolated miRNAs from EVs and free-circulating serum in both lung cancer patients and healthy controls. EVs were isolated from 500 µL of serum, while free-circulating miRNA was extracted from 200 µL of serum, and in both cases, RNA was eluted in 30 µL of buffer. The average concentration of EV-derived miRNA in lung cancer patients was 78.53 ± 55.74 ng/µL, compared to 16.78 ± 8.82 ng/µL in free-circulating serum. Among the healthy controls, EV miRNA concentration averaged 69.61 ± 48.46 ng/µL, while free-circulating miRNA measured 12.36 ± 5.61 ng/µL. When normalized to serum input volume, these correspond to 4.71 ng/µL serum for patient-derived EV miRNA and 2.39 ng/µL serum for free serum miRNA (Figure 3a). Similarly, in healthy controls, EV miRNA yielded 4.18 ng/µL serum, while the free-circulating fraction yielded 1.85 ng/µL serum (Figure 3b). These findings confirm a consistent and significant enrichment of miRNAs in the EV compartment compared to the free-circulating serum fraction in both patients and healthy individuals.

Interestingly, the amount of miRNA in the patient EV samples was considerably higher compared to the miRNA content of EVs from healthy controls. However, the data indicates that there was no significant difference between the miRNA concentrations in the EVs of patients with lung cancer and those of healthy individuals (*p* = 0.76), nor between the miRNA concentrations in the serum of patients and those of healthy individuals (*p* = 0.39).

### 3.5. Dysregulation of Several EVs miRNA and Circulating miRNA in Lung Cancer Patients

Previous studies have reported that multiple miRNAs show abnormal expression patterns in patients with lung cancer. Here, we conducted qRT-PCR for one tumor suppressor miRNA, Let-7a-5p, and one oncogenic miRNA, miR-21-3p, in EVs derived from 37 patients with lung cancer and compared them with 37 healthy individuals. miR-103a-3p was used as an endogenous control for normalization. In addition, we evaluated the circulating miRNAs in the serum of all patients and healthy individuals.

**Figure 4:**
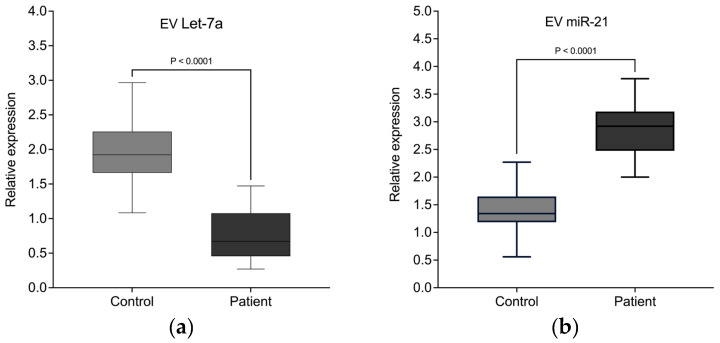
EVs miRNAs differentially expressed in lung cancer patients. Quantitative real-time PCR analysis of differentially expressed miRNAs in serum EVs from patients with lung cancer versus healthy controls. Let-7a-5p (**a**) and miR-21-3p (**b**) reached statistical significance.

The results indicate, as shown in Table 3, that the expression levels of EVs Let-7a-5p (Figure 4a) was downregulated in lung cancer patients compared to the controls, while the expression of EVs miR-21-3p (Figure 4b) was significantly upregulated in lung cancer patients compared to the controls.

**Figure 5:**
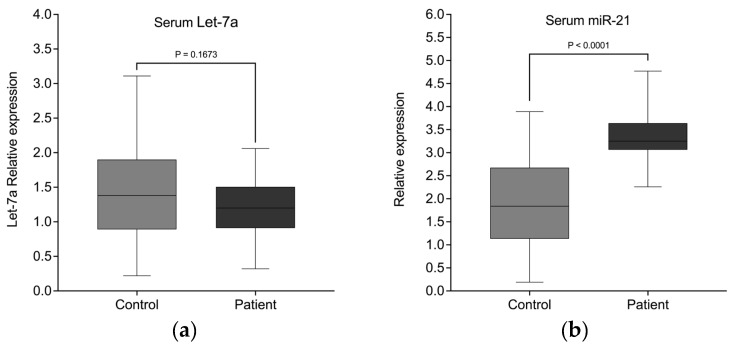
Circulating miRNAs differentially expressed in lung cancer patients. Quantitative real-time PCR analysis of differentially expressed miRNAs in serum EVs from patients with lung cancer versus healthy controls. Only hsa-miR-21-3p (**b**), but not hsa-Let-7a (**a**) reached statistical significance.

Analysis of the circulating miRNA expression levels in serum (Table 4) was not able to detect the differences in Let-7a-5p expression between patients and HC as identified in EV miRNA. In total serum RNA, both groups maintained comparable expression of hsa-let-7a-5p, indicating that Let-7a-5p is efficiently targeted in EVs (Figure 5a). In contrast, the significantly elevated miR21-3p expression in patient serum is also observed when analyzing total serum RNA. (Figure 5b).

Interestingly, when comparing EV-associated and total serum miRNA levels within each group, we observed distinct distribution patterns for the two miRNAs. hsa-let-7a-5p was predominantly detected in the EV fraction, with minimal levels in the total serum in both patients and controls, indicating preferential encapsulation of this miRNA in EVs. In contrast, hsa-miR-21-3p was present in both EVs and total serum at comparable levels, suggesting that a substantial proportion of this miRNA circulates outside EVs as free miRNA, in addition to its EV-associated form.

### 3.6. Association of miRNA Expression with Clinicopathological Features

The expression levels of Let-7a-5p and miR-21-3p in EVs and the serum were analyzed with respect to different clinicopathological features of patients with lung cancer.

There was no statistically significant difference in the expression of Let-7a-5p (Table 5) in the EVs or serum of patients with lung cancer with respect to age group (<60 vs. ≥60 years) or gender (male vs. female subjects), with *p*-values > 0.05, as well as for healthy controls. Nevertheless, the expression levels of Let-7a-5p in EVs were downregulated in tumor patients at advanced stages (Stage III–IV) when compared with the expression levels found in early-stage patients (Stage I–II) (*p* < 0.05), though they remained unaltered in the serum. However, Let-7a-5p expression levels did not significantly differ between adenocarcinoma and squamous cell carcinoma (*p* > 0.05).

Conversely, hsa-miR-21-3p (Table 6) was significantly upregulated in the EVs and serum from lung cancer patients in some clinicopathological features. Its expression in EVs was significantly higher in patients with advanced tumor stages (Stage III–IV) compared to patients with cancer in the early stages (Stage I–II) (*p* < 0.01). In addition, the levels of hsa-miR-21-3p were higher in adenocarcinoma patients compared to those with squamous cell carcinoma (*p* < 0.05). No significant associations between hsa-miR-21-3p expression levels and smoking status, age, or gender were detected (*p* > 0.05).

To specifically address the diagnostic utility of these two microRNAs at the early stages of lung cancer, we compared expression levels between early-stage NSCLC (Stage I–II) patients and healthy controls. No statistically significant differences were observed in either the serum or EVs.

## 4. Discussion

The majority of lung cancer patients are diagnosed at advanced stages, frequently leading to an unfavorable prognosis. This has driven researchers to develop biomarkers that can detect lung cancer at an early stage and improve the accuracy of diagnosis and treatment. In recent years, miRNAs have been discovered to play a crucial role in the pathogenesis and progression of lung cancer. Consequently, many research groups have focused on optimizing the potential of miRNAs as biomarkers for lung cancer [22].

Since miRNAs are involved in multiple cellular mechanisms that regulate tumor growth and progression, their use as biomarkers identified in blood has yielded very promising outcomes in the early detection of cancer. This is due to their relative stability against external factors such as pH alternation, storage, freezing, and thawing, as well as their resistance to degradation by endonucleases [23]. However, the results are sometimes contradictory, with variability in miRNA detection and inconsistent findings across different studies. This highlights the need for more reliable and consistent biomarkers for lung cancer detection.

To address these challenges, researchers are increasingly turning to EVs as an alternative source for miRNA as biomarkers. EVs are lipid bilayer nanovesicles that play a critical role in cell-to-cell communication by transporting bioactive molecules such as proteins, lipids, and nucleic acids, including miRNAs [24].

The miRNAs within EVs offer several advantages over free-circulating miRNAs. The miRNA within EVs is protected from degradation, thereby enhancing the stability and reliability of miRNA detection in bodily fluids. In addition, EVs are constantly released by living cells and are present in various body fluids, including the blood, urine, and saliva, making them accessible for non-invasive diagnostic tests. Most importantly, the miRNA profiles of EVs can reflect the molecular changes occurring within tumors, providing a more accurate snapshot of the disease state [6,18]. Despite this promising background, only a few studies have directly compared EVs and free circulating miRNAs in lung cancer.

In the present study, we isolated miRNAs from EVs and free-circulating miRNAs from serum in both lung cancer patients and healthy individuals. EV-derived miRNAs from lung cancer patients were obtained at an average of 471.2 ng per 100 µL serum, while the corresponding free-circulating fraction yielded 251.7 ng per 100 µL serum. Similarly, in healthy controls, EV-associated miRNAs were obtained at 417.7 ng per 100 µL serum, compared to 185.4 ng per 100 µL serum in the free-circulating component. These findings confirm a consistent enrichment of miRNAs in the EV compartment compared to free-circulating serum across both the cancer and control groups. This pattern aligns with previous studies [3]. For example, Dohmen et al. reported higher miRNA concentrations in exosomes versus serum in colorectal cancer patients (1504 ng/100 µL vs. 1024 ng/100 µL, respectively) [25]. Although the absolute concentrations in our lung cancer cohort are lower, the proportional enrichment in EVs is consistent, supporting the hypothesis that EVs provide a protective environment against serum RNase-mediated degradation.

The expression levels of miRNAs in cancer vary significantly, with both oncogenic and tumor-suppressive miRNAs being differentially regulated. Depending on the function of the miRNA, both variants can be beneficial for the tumor, inducing upregulation of miRNAs that act as proto-oncogenes, promoting tumor growth, and downregulation of miRNAs that act as tumor suppressors [26]. Based on this rationale, we analyzed an oncogenic and a tumor-suppressive miRNA in EVs derived from lung cancer patients, both previously reported as dysregulated in lung cancer patients [1].

In the present study, we found that the tumor suppressor miRNA Let-7a-5p is significantly downregulated in EVs derived from lung cancer patients compared to healthy individuals. In contrast, its abundance in free-circulating serum miRNA did not differ between groups. The downregulation of this miRNA in EVs isolated from lung cancer patients aligns with prior studies reporting reduced Let-7a-5p levels in EVs from lung [13], breast [27], and colorectal cancer [28].

However, the comparable expression levels of Let-7a-5p in the serum of both lung cancer patients and healthy controls in our present study contradicts previous findings that reported upregulation of this miRNA in the serum [29] of lung cancer patients. This discrepancy may be attributed to selective miRNA packaging mechanisms in EVs, which are regulated by specific RNA-binding proteins such as hnRNPA2B1 and YBX1 that recognize sequence motifs or secondary structures and direct miRNAs into EVs [30,31]. This selective encapsulation can lead to altered EV miRNA profiles independent of total serum miRNA levels. Furthermore, tumor cells may preferentially load tumor-suppressive miRNAs, such as let-7a, into EVs for secretion as a means of reducing their intracellular tumor-suppressive effects, while maintaining a balanced overall level in circulation contributed by non-tumor cells [32].

Multiple studies have reported the upregulation of miR-21-3p in various malignancies, both as free-circulating and EV-associated miRNA. Our data support these findings, demonstrating increased miR-21-3p levels in EVs isolated from the serum of patients with lung cancer [33] and free-circulating serum miR-21-3p [34]. Elevated miR-21 expression levels have been linked to tumor development, growth, and size through suppression of the tumor suppressor PTEN. This suggests the presence of miR-21 at early disease stages and thereby supports its potential utility in early detection.

Although previous studies have reported that both Let-7a-5p and miR-21-5p may serve as promising biomarkers for early-stage NSCLC detection due to their differential expression compared to healthy individuals [35,36], our findings did not reveal a statistically significant difference in the expression levels of these miRNAs between early-stage NSCLC patients (Stage I–II) and healthy controls. This lack of significance may be attributed to the limited number of Stage I patients in our cohort, reducing statistical power, as well as possible heterogeneity among early-stage disease presentations. Additionally, it is plausible that these two miRNAs alone are not sufficient for early detection, and a panel of multiple miRNAs or additional biomarkers might be required for improved sensitivity and specificity. Nevertheless, the overall trend of downregulation of Let-7a and upregulation of miR-21 in NSCLC patients relative to healthy controls aligns with previous reports and supports their utility in diagnosis and prognosis, particularly in more advanced stages [37,38].

### Limitations

This study has some limitations. The cohort size was relatively small (*n* = 37 per group), which may limit the statistical power and generalizability of our findings. Furthermore, the absence of an independent validation cohort and the lack of blinded analysis restrict the extent to which the diagnostic potential of EV-associated hsa-miR-21-3p can be confirmed at this stage. Therefore, while our data suggest that EV-derived hsa-miR-21-3p is a promising biomarker for NSCLC detection, future studies with larger, independent, and blinded cohorts will be required to validate and extend these observations.

## 5. Conclusions

In conclusion, we were able to validate recent research findings regarding cancer miRNA enrichment in circulating EVs in contrast to the serum. EV levels of Let-7a-5p and miR-21-3p were significantly different in patients with lung cancer when compared with healthy controls. While free-circulating miR-21 levels were significantly increased in lung cancer patients compared to healthy controls, these differences were not observed for free-circulating Let-7a. Altogether, these findings support the potential of specific EV-miRNA cargo analysis as a minimally invasive approach in NSCLC detection and monitoring.

## Figures and Tables

**Table 1:** Primers used in this study.

Product Name	Sequence	GeneGlobe Id
hsa-let-7a-5p	5′UGAGGUAGUAGGUUGUAUAGUU	YP00205727
hsa-miR-21-3p	5′CAACACCAGUCGAUGGGCUGU	YP00204302
hsa-miR-103a-3p	5′AGCAGCAUUGUACAGGGCUAUGA	YP00204063
UniSp6		YP00203954

**Table 2:** The characteristics of the participants.

Groups		Patients		Healthy Control	
		No. 37	%	No. 37	%
Age	<60	10	27.03	16	43.24
	>60	27	72.97	21	56.76
Gender	Male	22	59.46	24	64.86
	Female	15	40.54	13	35.14
Smoking	Non-Smokers	17	45.95	15	40.54
	Former smokers	15	40.54	9	24.32
	Current smokers	5	13.51	13	35.14
TNM stages	Stage I	8	21.62		
	Stage II	14	37.84		
	Stage III	9	24.32		
	Stage IV	6	16.22		
Type of Cancer	AD *	31	83.78		
	SCC *	6	16.22		
EGFR statue	EGFR wild type	32	86.49		
	EGFR mutant	5	13.51		

* AD: adenocarcinoma, SCC: squamous cell carcinoma.

**Table 3:** EVs miRNAs differentially expressed in patients and healthy controls.

Groups	Patients EVs		Control EVs		*p* Value
	Mean	SD	Mean	SD	
hsa-let-7a-5p	0.76	0.35	1.96	0.46	0.0001
hsa-miR-21-3p	2.87	0.47	1.41	0.39	0.0001

**Table 4:** Circulating miRNAs differentially expressed in patients and healthy controls.

Groups	Patients Serum		Control Serum		*p* Value
	Mean	SD	Mean	SD	
hsa-let-7a-5p	1.21	0.41	1.40	0.72	0.16
hsa-miR-21-3p	3.29	0.51	1.92	0.91	0.0001

**Table 5:** Expression levels of Let-7a-5p in EVs and serum across clinicopathological features of lung cancer patients.

Clinicopathological Features	EVs (Mean ± SD)	*p* Value	Serum (Mean ± SD)	*p* Value
Age (<60 vs. ≥60)	0.72 ± 0.12	>0.05	1.12 ± 0.44	>0.05
Gender (Male vs. Female)	0.70 ± 0.13	>0.05	1.14 ± 0.46	>0.05
Smoking (Current Smokers vs. Non-smokers/Former Smokers)	0.70 ± 0.14	>0.05	1.15 ± 0.42	>0.05
TNM Stages (Stage III–IV vs. Stage I–II)	0.67 ± 0.13	<0.05	1.16 ± 0.45	<0.05
Type of Cancer (AD vs. SCC)	0.71 ± 0.15	>0.05	1.13 ± 0.43	>0.05

**Table 6:** Expression levels of miR-21-3p in EVs and serum across clinicopathological features of lung cancer patients.

Clinicopathological Features	EVs (Mean ± SD)	*p* Value	Serum (Mean ± SD)	*p* Value
Age (<60 vs. ≥60)	2.85 ± 0.35	>0.05	3.08 ± 0.41	>0.05
Gender (Male vs. Female)	2.80 ± 0.30	>0.05	3.12 ± 0.39	>0.05
Smoking (Current Smokers vs. Non-smokers/Former Smokers)	2.70 ± 0.25	>0.05	3.10 ± 0.37	>0.05
TNM Stages (Stage III–IV vs. Stage I–II)	2.74 ± 0.33	<0.01	3.11 ± 0.43	<0.01
Type of Cancer (AD vs. SCC)	2.92 ± 0.32	<0.05	3.18 ± 0.33	<0.05

## Data Availability

The datasets used and/or analyzed during the current study are available from the corresponding author on reasonable request.
